# An approach for the measurement of the bulk temperature of single crystal diamond using an X-ray free electron laser

**DOI:** 10.1038/s41598-020-71350-x

**Published:** 2020-09-03

**Authors:** A. Descamps, B. K. Ofori-Okai, K. Appel, V. Cerantola, A. Comley, J. H. Eggert, L. B. Fletcher, D. O. Gericke, S. Göde, O. Humphries, O. Karnbach, A. Lazicki, R. Loetzsch, D. McGonegle, C. A. J. Palmer, C. Plueckthun, T. R. Preston, R. Redmer, D. G. Senesky, C. Strohm, I. Uschmann, T. G. White, L. Wollenweber, G. Monaco, J. S. Wark, J. B. Hastings, U. Zastrau, G. Gregori, S. H. Glenzer, E. E. McBride

**Affiliations:** 1 SLAC National Accelerator Laboratory, Menlo Park, CA 94025 USA; 2 Aeronautics and Astronautics Department, Stanford University, Stanford, CA 94305 USA; 3 European X-Ray Free-Electron Laser Facility GmbH, Holzkoppel 4, 22869 Schenefeld, Germany; 4 Atomic Weapons Establishment, Aldermaston, Reading, RG7 4PR UK; 5 Lawrence Livermore National Laboratory, Livermore, CA 94550 USA; 6 Centre for Fusion, Space and Astrophysics, Department of Physics, University of Warwick, Coventry, CV4 7AL UK; 7 Department of Physics, Clarendon Laboratory, University of Oxford, Parks Road, Oxford, OX1 3PU UK; 8 Institut für Optik und Quantenelektronik, Friedrich-Schiller-Universität Jena, Max-Wien-Platz 1, 07743 Jena, Germany; 9 Helmholtz-Institut Jena, Fröbelstieg 3, 07743 Jena, Germany; 10 School of Mathematics and Physics, Queen’s University, University Road BT7 1NN, Belfast, UK; 11 Institut für Physik, Universität Rostock, A.-Einstein-Str. 23-24, 18059 Rostock, Germany; 12 Deutsches Elektronen Synchrotron, Notkestrasse 85, 22607 Hamburg, Germany; 13 University of Nevada, Reno, NV 89557 USA; 14 Dipartimento di Fisica, Università di Trento, Via Sommarive 14, 38123 Povo, TN Italy

**Keywords:** Condensed-matter physics, Techniques and instrumentation, Materials science, Physics

## Abstract

We present a method to determine the bulk temperature of a single crystal diamond sample at an X-Ray free electron laser using inelastic X-ray scattering. The experiment was performed at the high energy density instrument at the European XFEL GmbH, Germany. The technique, based on inelastic X-ray scattering and the principle of detailed balance, was demonstrated to give accurate temperature measurements, within $8\%$ for both room temperature diamond and heated diamond to 500 K. Here, the temperature was increased in a controlled way using a resistive heater to test theoretical predictions of the scaling of the signal with temperature. The method was tested by validating the energy of the phonon modes with previous measurements made at room temperature using inelastic X-ray scattering and neutron scattering techniques. This technique could be used to determine the bulk temperature in transient systems with a temporal resolution of 50 fs and for which accurate measurements of thermodynamic properties are vital to build accurate equation of state and transport models.

## Introduction

From the thermal shield of a spacecraft during atmospheric re-entry^1^ to the interior of Jovian planets^2^, matter is often found at pressures and temperatures that are at the limits of where conventional condensed matter and plasma physics formalisms are valid^3^. At such extreme conditions, the kinetic energy of the electrons is comparable to the potential energy of interaction between electrons and the nuclei. For these systems, direct and accurate measurements of thermodynamic and transport properties are vital. Extreme states of matter can be produced by dynamic laser-driven compression^4–6^ and laser heating techniques^7,8^. These methods can excite a material into a transient state of simultaneously high density and temperature, and thereby enable access to previously unexplored regions of phase space. State-of-the-art experiments investigate the properties of materials driven into these conditions by coupling high-energy lasers with suitable probing techniques^9,10^. In particular, X-ray scattering has proven to be a powerful tool for determining the structure and density, and the development of X-ray free electron lasers (XFELs) has led to improved understanding of materials under dynamic compression conditions^11–18^. While it is possible to directly investigate the structure of a material under dynamic compression, measuring the temperature remains a challenge. In these experiments, the temperature has typically been estimated from hydrodynamic simulations or inferred through the use of streaked optical pyrometry, which measures the thermal self-emission of the surface of a material. However, this technique is only valid at temperatures in excess of 4,000 K^19,20^ and relies on assumptions of the behaviour of emissivity at extreme conditions. Extended X-ray absorption fine structure measurements (EXAFS) have been made to investigate simultaneously the density and temperature in compressed matter^21^. However, this technique requires a knowledge of the pressure dependent Debye temperature. Diffraction-based Debye Waller measurements have also been proposed as a temperature diagnostic for compressed matter, and have been demonstrated from ultrafast-heated gold using MeV electron diffraction techniques^22^. A solution to this temperature measurement problem may arise through the use of inelastic X-ray scattering to measure the dynamic structure factor, $S(Q, \omega )$. Inelastic X-ray scattering with electronvolt to kilo-electronvolt resolution (i.e. Thomson scattering of “free” electrons) has been well-established for determining the Fermi temperature, densities and ionisation state of dynamically compressed matter, and has been demonstrated at both large laser facilities^23,24,25,26^ and at hard XFELs^27,28^. By making use of milli-electronvolt (meV) resolution inelastic X-ray scattering, one can measure changes in the X-ray photon energy due to scattering from collective excitations such as phonons in a solid^29–32^, acoustic waves in a liquid^33–35^ or ion acoustic modes in the warm dense matter regime^36,37^. By probing these low-energy collective atomic/ionic motions and employing the principle of detailed balance^38^, one can directly infer the temperature of the atomic subsystem in thermal equilibrium. Here, we describe the use of the European XFEL^39–42^ to measure the dynamic structure factor of single crystal (100) diamond at room temperature and at 500 K, with meV resolution. The lattice temperature is determined from the asymmetry of the measured inelastic scattering spectra and found to be within $8\%$ of that measured by a thermocouple attached to the sample holder, comparable to previous measurements conducted at third generation synchrotron facilities^43^. We describe the experimental setup, and present the approach to process the data and subsequently extract temperatures. By taking full advantage of the XFEL, this technique could be used to make time-resolved temperature measurements of transient, laser driven states of matter.

## Experimental details

**Figure 1:**
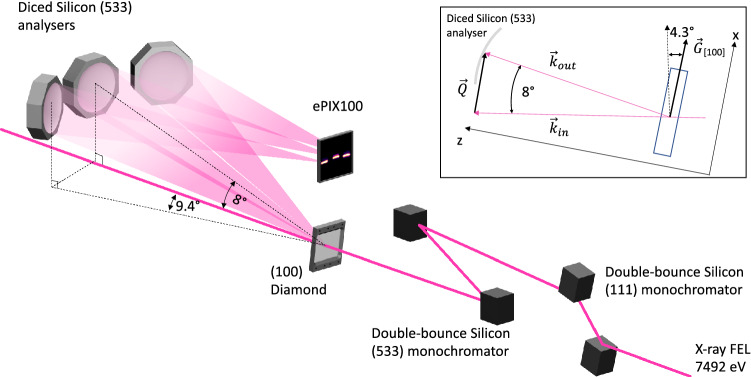
Sketch of the experimental setup used to perform millielectronvolt inelastic X-ray scattering at the HED end-station at the European XFEL^44^, following the proof of principle of McBride et al. ^5^. Incident X-ray pulses at $7{,}492\;\mathrm{eV}$ are first monochromatised using a succession of two double-bounce silicon monochromators arranged in a dispersive configuration. Monochromatised X-ray pulses are then incident on a diamond sample oriented such that the scattering vector $\vec {Q}$ is parallel to the [100] crystallographic direction of diamond. The inset shows a side view of the experimental configuration with the orientation of the different parts. Scattered photons are finally collected by the three diced silicon analysers and finally focused on an ePIX100 detector^48^.

The experiments were performed at the high energy density (HED) instrument at the European XFEL GmbH, Germany. The experimental setup is shown in Fig. 1, and is described in detail by Wollenweber et al.^44^ The self-amplified spontaneous emission (SASE) X-ray beam mode delivered single pulses with $10^{12}$ X-ray photons at a repetition rate of 10 Hz^45^. The incident X-ray energy was chosen to be 7,492 eV with a bandwidth of $\frac{\Delta E}{E}=10^{-3}$ to ensure a near backscattering ($\sim 87.5^{\circ }$) reflection on the high-resolution monochromator and the spherically diced analyser^46^. The X-ray beam was first monochromatised to a bandwidth of $\frac{\Delta E}{E}=10^{-4}$ using a symmetric silicon (111) monochromator in a (+-) non-dispersive configuration, and further monochromatised using a high-resolution symmetric silicon (533) monochromator in a (-+) non-dispersive near backscattering configuration to achieve a bandwidth $\frac{\Delta E}{E}=4\times 10^{-6}$. After the monochromatisation, the photon number per pulse falls to $10^{9}$ as calculated from dynamical X-ray diffraction theory^47^. Beryllium compound refractive lenses, $9 \, \mathrm{m}$ upstream of the sample, were used to focus the X-ray pulses. To ensure a large probing volume, the sample was positioned out of the best focus plane resulting in an X-ray spot size of 25 $\upmu$m.

The focussed and monochromatised X-ray beam was incident onto a 250 $\upmu$m thick sample of single crystal diamond from Applied Diamond, Inc., with the surface oriented parallel to the [010] direction. This thickness corresponds to half the attenuation length of the 7,492 eV X-ray photons in diamond, and was chosen to balance the scattering signal and the broadening of the measured spectra due to sample size effects^46^. In order to minimise quasi-elastic scattering that one would observe in the presence of disorder e.g. grain boundaries, defects, the measurement was made on high-purity single crystal diamond. The measurement was performed on both room temperature and heated diamond at 500 K using a resistive heater. Details of the resistive heater are described in the “Methods” section. Photons scattered from the sample were energy dispersed by three diced single crystal silicon (533) analysers with a cube size of $1.65\,\mathrm{mm}\times 1.65\,\mathrm{mm}$. The analysers were arranged in a Johann geometry with a 1 m diameter^46^. The three analysers sit on a common horizontal rail free to move in the vertical direction such that the side analysers are horizontally offset by $9.4^{\circ }$ with respect to the central analyser. The central analyser was positioned at an angle of $8^{\circ } \pm 1^{\circ }$ and the sample was vertically tilted by an angle of $4.3^{\circ }$ towards the monochromator in order to align the scattering vector $\vec {Q} = \vec {k}_{out} - \vec {k}_{in}$ with the [001] direction within the first Brillouin zone of diamond, and hence to be mostly sensitive to the longitudinal acoustic mode along this direction. For this geometry, the analysers collected photons with momentum transfer of $||\vec {Q}|| = 0.52 \pm 0.1\,{\AA }^{-1}$ for the central analyser and $||\vec {Q}|| = 0.81 \pm 0.1\,{\AA }^{-1}$ for the side analysers. The uncertainty on the momentum transfer arises from the distribution of scattering angles collected by the 10 cm diameter spherical diced analysers and corresponds to the $67\%$ confidence interval.

Photons which are both energy dispersed and focused using the diced silicon crystal analysers were detected using an ePIX100 detector with a pixel size of 50 $\upmu$m $\times$ 50 $\upmu$m^48^. The energy resolution of the entire system is dominated by the intrinsic energy resolution of the (533) reflection of silicon ($\sim$ 33 meV) and the different geometrical contributions to its broadening i.e. Johann aberration ($\sim$ 4 meV), pixel contribution ($\sim$ 7.5 meV), and sample size effects^49^. This was measured at room temperature using a 500 $\upmu$m-thick sample of polymethyl methacrylate (PMMA), as the scattering from this amorphous material is dominated by quasi-elastic scattering. In the heated case, a 250 $\upmu$m-thick piece of amorphous $\hbox {SiO}_2$ was used as a calibrant for the energy resolution, as the melting point of PMMA is around 430 K.

**Figure 2:**
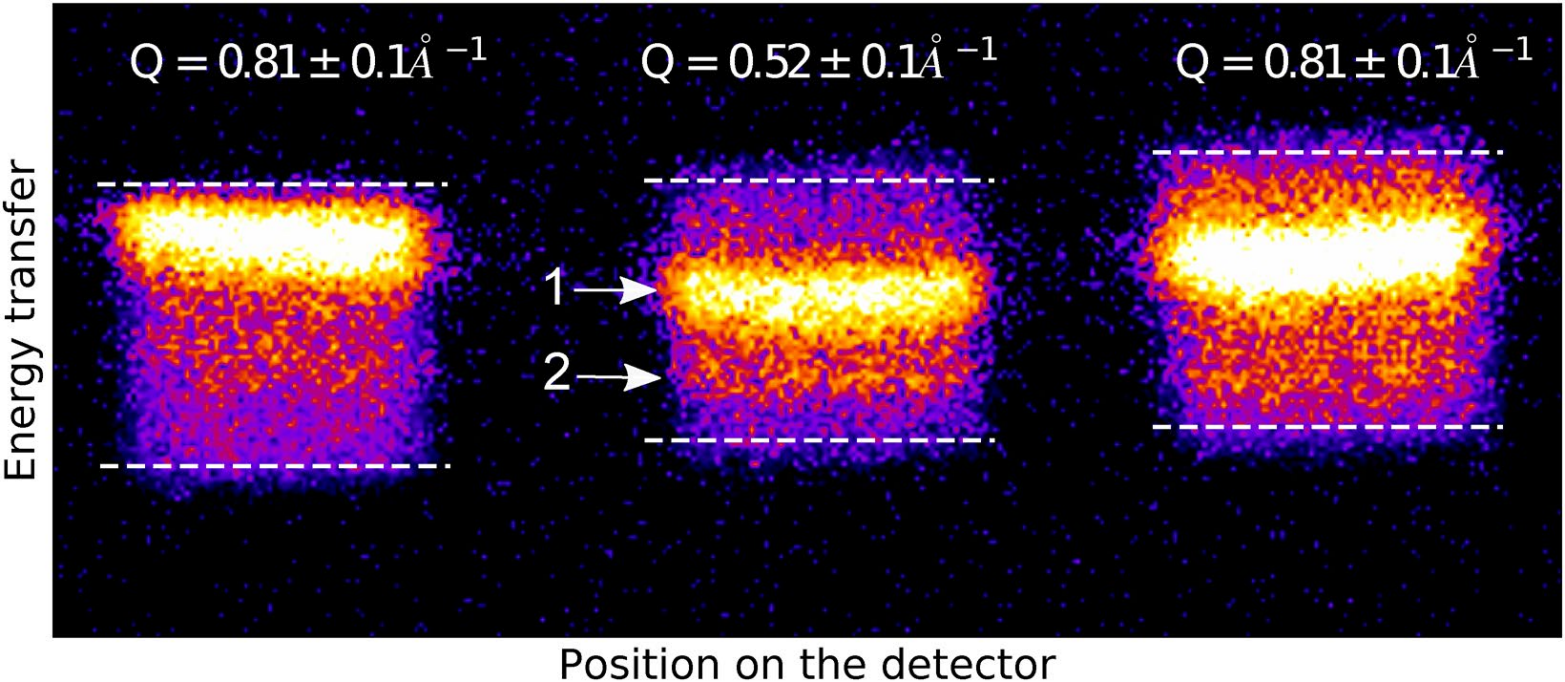
Overlay of two raw images corresponding to the collected photons scattered from 500 $\upmu$m-thick PMMA (square) and 250 $\upmu$m-thick diamond at room temperature (bright lines) on all three analysers. The squares show the focused images of each analyser on the detector and were obtained using the non-monochromatised X-ray beam. The brighter regions correspond to the raw data collected on room temperature diamond and was obtained using the monochromatised X-ray beam. The vertical direction on the image corresponds to the energy dispersive direction. One can observe on each square a bright band corresponding to the Stokes line (arrow 1) and a dimmer band under it corresponding to the anti-Stokes line (arrow 2). The horizontal white dashed lines represent the boundaries of the image of the analysers on the ePIX100 detector and correspond to the vertical dark dashed lines in Fig. 3. It should be mentioned that the intensity tails outside the white dashed lines, and clearly visible in the intensity fall off beyond the vertical dashed lines in Fig. 3, are most likely caused by defocusing effects.

The resolution of each analyser at room temperature, and following resistive heating, was obtained using the data processing procedure described below, and determined by fitting a pseudo-Voigt profile to the obtained quasi-elastic scattering spectra. The experimental data along with their best fit are shown in Fig. 1 in the Supplemental Material. The energy resolutions for each analyser are summarised in Table 1, and examples of the raw data are shown in Fig. 2.

**Table 1:** Measured energy resolution for the three analysers using 500 $\upmu$m-thick PMMA at room temperature and 250 $\upmu$m-thick $\hbox {SiO}_2$ at 500 K.

	$T=294 \pm 2$ K (meV)	$T=503 \pm 8$ K (meV)
$Q = 0.81 \pm 0.1{\AA }^{-1}$	74	68
$Q = 0.52 \pm 0.1{\AA }^{-1}$	67	59
$Q = 0.81 \pm 0.1{\AA }^{-1}$	76	64

The smaller values for $\hbox {SiO}_2$ at 500 K are attributed to a better alignment of the spectrometer when the heated data were collected. The energy difference between the two samples translate to a $3\%$ error in the measured temperature.

## Data processing

For each X-ray pulse, the scattered photons are collected by the spherically diced analyser and are focused on the detector. Given the small inelastic cross section, a single photon counting routine is used to classify each pixel on the detector as a single photon depending on its count value and the count values of the surrounding pixels. The procedure is further discussed in the Supplemental Materials. Example raw data are presented in Fig. 2 and correspond to the summation of the single photons scattered from room temperature diamond at 10 Hz using 95,547 X-ray pulses.

**Figure 3:**
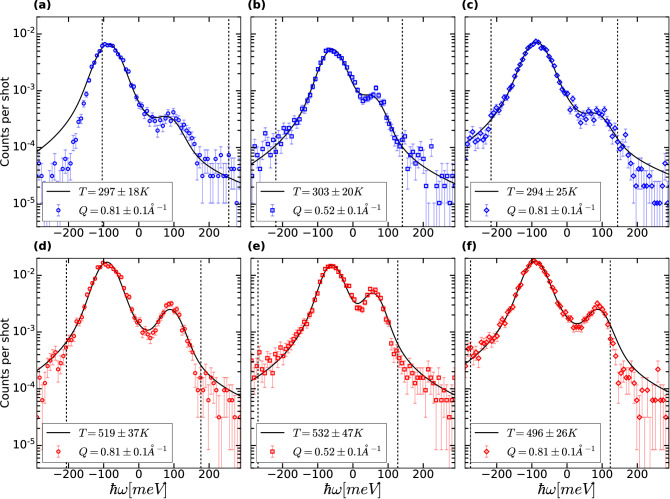
Inelastic spectra for each analyser on room temperature (100) single crystal diamond (**a**–**c**) and resistively heated (100) single crystal diamond to $503 \pm 8$ K (**d**–**f**) showing normalised counts per shot as a function of energy transfer $\hbar \omega$. Traces on the left, middle and right correspond to data obtained from the left, central and right analysers in Fig. 1. The spectra are fitted using Eq. (1) (solid black line) within the energy range defined by the vertical black dashed lines. Temperature is then measured from the intensity asymmetry between the positive and negative energy sides by the use of the detailed balance principle. The inelastic components for the centre analyser in the cold case are shown in Fig. 2 in the Supplemental Materials.

The subsequent spectra are then unwrapped to correct for the curvature of the image of the analysers on the detector. This is done by fitting a quadratic function to each trace in Fig. 2 in order to find a relationship between the non-dispersive axis and the energy dispersive axis. Each pixel is then adjusted based on the fitted relation to obtain a linear trace. The curvature-corrected trace is then integrated along the horizontal direction (non-dispersive direction) to give the experimental data points shown in Fig. 3. The energy transfer is finally constructed from the energy per pixel relation by measuring the displacement of the quasi-elastic component along the energy dispersion axis with the incident X-ray energy and by indexing the zero energy transfer for each analyser with the position of the maximum intensity of the corresponding instrument function.

## Fitting procedure and model

The detected scattered intensity, following the data processing described above, can be expressed as the sum of the elastic coherent double differential cross section and the inelastic coherent one-phonon double differential cross section^50^. Using the second quantisation of the vibrational modes in a solid in terms of the phonon creation and annihilation operators^50,51^ and assuming thermal equilibrium, one can express the scattered intensity from phonon modes in single crystal diamond in the first Brillouin zone using Eq. (1). The derivation of this expression is presented in the Supplemental materials.

1 $$\begin{aligned} I(\hbar \omega )={} R(\hbar \omega , \vec {q}) *\left( \dfrac{I_0}{\pi } \dfrac{\Gamma _{0}(\vec {q})}{\left( \hbar \omega )^2+\Gamma _{0}(\vec {q}\right) ^2} \right. \nonumber \\+\left. \langle n\left( \omega \right) \rangle \sum _\lambda \dfrac{I_\lambda }{\pi } \left[ -\dfrac{\Gamma _{\lambda }(\vec {q})}{\left( \hbar \omega +\hbar \omega _{\lambda }(\vec {q})\right) ^2+\Gamma _{\lambda }(\vec {q})^2} +\dfrac{\Gamma _{\lambda }(\vec {q})}{\left( \hbar \omega -\hbar \omega _{\lambda }(\vec {q})\right) ^2+\Gamma _{\lambda }(\vec {q})^2}\right] \right) . \end{aligned}$$

The first Lorentzian accounts for the elastic scattering contribution to the recorded intensity and the summation over $\lambda$ for the inelastic scattering intensity arises from the different phonon branches, $\lambda$, in the material. $\vec {q}$ is the phonon wavevector defined within the first Brillouin zone. $\omega _{\lambda }(\vec {q})$ is the phonon frequency of the branch $\lambda$ at wavevector $\vec {q}$. $\langle n(\omega _{\lambda }(\vec {q}))\rangle$ is the Bose occupation number of phonons with frequency $\omega _{\lambda }(\vec {q})$. It is defined as $\langle n(\omega )\rangle = \big [e^{\hbar \omega \beta }-1\big ]^{-1}$ and $\beta = \frac {1}{k_B T}$. Finally, $\hbar \omega$ is the energy exchanged during the scattering event and is counted positive when the photon gains energy during the interaction.

$R(\hbar \omega , \vec {q})$ is the instrument function as determined by measuring the inelastic scattering spectrum of 500 $\upmu$m of PMMA for room temperature, and 250 $\upmu$m $\hbox {SiO}_2$ at 500 K, see Table 1. In Eq. (1), T, $I_0$, $I_\lambda$, $\Gamma _{\lambda }(\vec {q})$, $\Gamma _{0}$, $\omega _{\lambda }(\vec {q})$ are free parameters. To account for anharmonic effects such as phonon–phonon interaction that may arise from higher order terms in the Taylor expansion of the interatomic potential, Lorentzian line shapes are used to model the recorded intensity on the detector^51^. In principle, $\Gamma _{\lambda }$, the width of the Lorentzian line shape, accounts for the finite lifetime of the vibration mode $\lambda$. However, this width is expected to be small as anharmonic effects are negligible for temperatures $k_B T \ll \hbar \omega _m$^51^, where $\omega _m$ is the maximum phonon frequency of the material. In the case of diamond, $\hbar \omega _m \sim 160$ meV corresponding to a temperature around 1,900 K. Given the resolution of the instrument, no physical information can be extracted from the numerical value of the $\Gamma _{\lambda }$ obtained from the best fit of the spectra.

## Results and discussion

Figure 3 shows the processed, unwrapped, and integrated data from single crystal diamond at room temperature (Fig. 3a–c) and at 500 K (Fig. 3d–f). The vertical dashed lines indicate the edges of the focused image of the analysers on the detector along the dispersive direction (corresponding to the white dashed lines in Fig. 2). The solid black lines correspond to the best fit to the data, within the vertical dashed lines, using the model described above. The fitting procedure is done by minimising the $\chi ^2$ and is further described in the “Methods” section. In this work, negative energy transfer corresponds to red-shifted photons, and positive energy transfer corresponds to blue-shifted photons.

The best fit parameters for both the cold and hot spectra are summarised in Table 2 (Table 3) for the room temperature (resistively heated) diamond spectrum. It has to be noted that the intensity of the quasi-elastic component in Eq. (1) is given by $I_0 \Gamma _0$. From the values reported in these tables, one can notice that the quasi-elastic component is negligible in all cases as expected. In addition, the width of the inelastic components, $\Gamma _{\lambda }$, is on the order of the energy per pixel resolution of the measurement ($\sim 7.5$ meV per pixel) and corresponds to sub-pixel broadening of the line shape. As a result, no information concerning the phonon lifetime could be extracted from these measurements.

**Table 2:** Values of the free parameters in Eq. (1) obtained from $\chi ^2$ minimisation for the room temperature diamond.

	$\omega _\lambda$ (meV)	$I_0\times \Gamma _0$ (meV)	$I_\lambda$ (a.u)	$\Gamma _\lambda$ (meV)
$Q = 0.81 \pm 0.1{\AA }^{-1}$	$87 \pm 4$	$2.10^{-12}$	94	5
$Q = 0.52 \pm 0.1{\AA }^{-1}$	$59 \pm 1$	$2.10^{-7}$	85	7.8
$Q = 0.81 \pm 0.1{\AA }^{-1}$	$87 \pm 1$	$1.10^{-11}$	97	4.7

**Table 3:** Values of the free parameters in Eq. (1) obtained from $\chi ^2$ minimisation for the resistively heated diamond spectra.

	$\omega _\lambda$ (meV)	$I_0\times \Gamma _0$ (meV)	$I_\lambda$ (a.u)	$\Gamma _\lambda$ (meV)
$Q = 0.81 \pm 0.1{\AA }^{-1}$	$91 \pm 2$	$3.10^{-14}$	81	4.3
$Q = 0.52 \pm 0.1{\AA }^{-1}$	$60 \pm 2$	$2.10^{-8}$	64	8.4
$Q = 0.81 \pm 0.1{\AA }^{-1}$	$91 \pm 2$	$4.10^{-9}$	83	9.3

In general, all the phonon branches contribute, with different weights, to the intensity recorded on the detector. However, given the experimental configuration, the longitudinal acoustic mode is the main contributor to the intensity. Along high-symmetry directions, the phonon polarisation vector $\vec {\epsilon _{\lambda }}$ is either collinear to $\vec {Q}$ (longitudinal mode) or perpendicular to $\vec {Q}$ (transverse mode), with $\vec {Q} = \vec {k}_{out} - \vec {k}_{in}$. The overall intensity $I_{ \lambda }$, Supplementary Equation S11, is related to the selection rule $\frac {\vec {Q}\cdot \epsilon _{\lambda }(\vec {q})}{\hbar \omega _{\lambda }}$, from which the only non-zero terms involve the longitudinal acoustic mode. The experimental data presented in Fig. 3 are best explained using a single longitudinal acoustic mode convolved with the instrument resolution.

When comparing the spectra collected at room temperature (Fig. 3a–c) and upon heating (Fig. 3d–f), one can observe a change in the intensity ratio between the red-shifted and the blue-shifted lines. This intensity ratio is related to the temperature of the system through the principle of detailed balance. The measured temperatures are reported in Table 4, and compared directly to temperature as measured by a type K thermocouple attached directly to the target plate (see “Methods” section).

**Table 4:** Comparison between the experimental temperature measured using the principle of detailed balance and the reading from the thermocouple used to control the temperature of the sample holder with a resistive heater.

	Thermocouple (K)	Experimental data (K)	Thermocouple (K)	Experimental data (K)
$Q = 0.81 \pm 0.1{\AA }^{-1}$	$294 \pm 2$	$297 \pm 18$	$503 \pm 8$	$519 \pm 37$
$Q = 0.52 \pm 0.1{\AA }^{-1}$	$294 \pm 2$	$303 \pm 20$	$503 \pm 8$	$532 \pm 47$
$Q = 0.81 \pm 0.1{\AA }^{-1}$	$294 \pm 2$	$294 \pm 25$	$503 \pm 8$	$496 \pm 26$

The uncertainty corresponds to the $1\sigma$ level from the $\chi ^2$ fit of the data, assuming normally distributed residuals. The error for the thermocouple corresponds to the uncertainty on the temperature measurement using a type K thermocouple for the cold case while it corresponds to the maximum of the previous uncertainty and the temperature fluctuations from the PID controller in the heated case.

In addition to the change in the amplitude of the red-shifted/blue-shifted lines upon heating, the total number of photons per X-ray pulse scattered by each analyser also increases with temperature, from 0.5 counts per pulse at room temperature to 1.6 counts per pulse at 500 K. This can be understood by considering the dependency of the theoretical expression for the intensity $I_{\lambda }$ with temperature (See supplemental materials). Since the temperature of the system is low compared to the Debye temperature of diamond ($\sim 1900$ K)^55^, the thermal motion of the atoms is still well approximated using a harmonic potential. As a result the energy of the phonon mode, $\omega _{\lambda }$, is unchanged upon heating and the intensity scales with the Bose–Einstein distribution which is an increasing function of temperature. It has to be noted that the Debye–Waller factor is a decreasing function of temperature. While these two factors have opposite contributions, the Debye–Waller factor is negligible in this case as the measurement was made in the first Brillouin zone (small Q) and on diamond which has a high Debye temperature.

**Figure 4:**
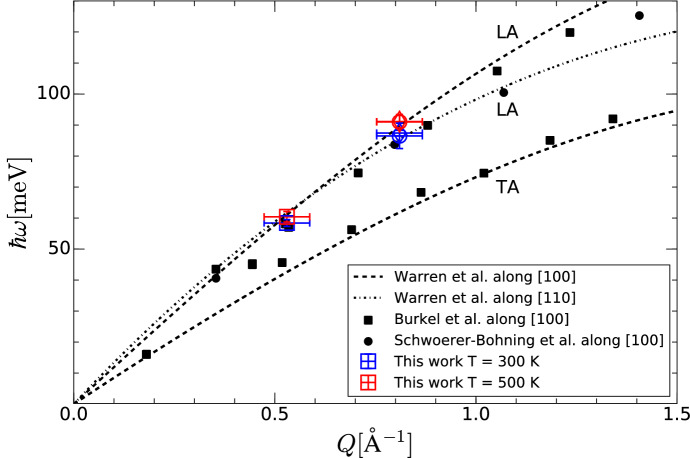
Measurement of the dispersion curve of single crystal (100) diamond. Open squares, circles, and diamonds correspond to data collected on the central, left and right analysers, respectively. Blue symbols correspond to room temperature data, and red symbols to the high-temperature data extracted from Fig. 3(a–f). The uncertainty along the momentum axis is arising from the finite dimension of the analyser which causes a distribution of scattering angles to be collected. Our measurements are compared with inelastic neutron and inelastic X-ray scattering data^52–54^. The dashed and dot-dashed lines correspond to neutron measurements along the [100] and [110] directions, respectively. X-ray scattering data are reported as the closed squares^53^ and closed diamonds^54^. The longitudinal and transverse modes are labelled as LA and TA.

Finally, the energy of the modes $\hbar \omega _\lambda$ extracted from our experimental measurements are compared with neutron scattering measurements^52^ and other inelastic X-ray measurements^29^, and are summarised in Fig. 4. As mentioned above, the side analysers are measuring along an intermediate direction between the [100] and [110]. As a result, the longitudinal acoustic modes corresponding to these two crystallographic directions are shown in Fig. 4. From this figure and the experimental configuration, the measured modes for the central analyser are consistent with the longitudinal acoustic modes of diamond as expected from the experimental design. Finally, to unambiguously determine the position of the modes for the side analysers, one would need to use a better energy resolution.

## Conclusions

High resolution (meV) inelastic X-ray scattering on room temperature single crystal (100) diamond and on resistively-heated single crystal (100) diamond up to $503 \pm 8$ K were performed at the high energy density (HED) instrument at the European XFEL GmbH, Germany using the SASE beam mode. The incoming X-ray beam at 7,492 eV was monochromatised using a combination of a (111) silicon channel cut crystal and a (533) silicon channel cut crystal. The scattered photons at specific reciprocal wave vectors were collected using three spherical diced (533) Si analysers and then focussed on an ePIX100 detector. From the asymmetry of the Stokes and anti-Stokes lines, the sample temperature was measured via the principle of detailed balance. This temperature was then compared with a thermocouple reading with a difference smaller than $8\%$ for single crystal diamond.

The method was tested on a resistively heated single crystal diamond for which no spatial and temporal gradient of thermodynamic quantities are expected. However, it has been previously reported in the literature that spatial gradients within the material could affect the temperature determination through the use of the principle of detailed balance^56,57^ and could be an obstacle, if not considered in the experimental design, to the use of this technique for transient states of matter. Several direct improvements are still worth mentioning. The seeded X-ray mode demonstrated at the Linac Coherent Light Source at SLAC National Accelator laboratory, California^28,58^ and expected in the future at European XFEL would provide a larger number of photons on the sample after the monochromator stage and thus reduce the number of X-ray pulses required to obtain a good quality spectrum. The energy resolution could also be improved by using higher order Bragg reflections for both the high-resolution monochromator and the spherical diced analysers. Finally large plasmas with reduced spatial gradients can be driven using energetic lasers for which this technique could be used^59^. Such improvements coupled with careful experimental design could offer an accurate temperature measurement for transient states of matter generated by dynamic laser compression and heating techniques which are expected to occur in the near future for instance the high-repetition rate (10 Hz) DiPOLE laser system at the HED instrument at the European XFEL GmbH, Germany.

## Methods

### Description of the sample heater

**Figure 5:**
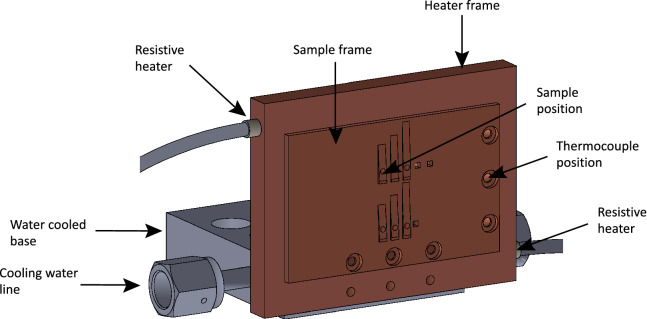
Schematic of the heater assembly used for the measurements at $503\pm 8$ K during the experiment at the high energy density end-station at the European XFEL, Hamburg, Germany.

A schematic of the assembly used to heat the samples is presented in Fig. 5. The assembly consisted of: (1) a copper sample plate; (2) a copper frame, which was heated by a pair of resistive heater cartridges (McMaster Carr) inserted into the sides of a copper frame; and (3) a water-cooled stainless steel base. Copper was chosen for the material of the heated elements due to its high thermal conductivity and high melting temperature. The sample plate was held against the copper frame by screws, and pressing the sample plate against the copper frame provided sufficient thermal contact for heating. The temperature of the sample plate was measured using a type K thermocouple (Accuglass). Power to the heater cartridges was controlled using a proportional–integral–derivative controller (Fuji Electric) which used a feedback loop to hold the sample plate at the desired temperature. The frame was screwed into the stainless steel water-cooled base with a 1/16-in. piece of MACOR ceramic inserted between them to help thermal isolation. A second thermocouple was placed on the water-cooled base to monitor its temperature. We observed that throughout the whole measurement over the course of several hours, the base plate was maintained at ambient temperature. For room temperature measurements, the heater was turned off and the thermocouple reading was confirmed to be 294 K during the data collection.

### Fitting procedure

The fitting procedure is done by minimizing the $\chi ^2$ error between the experimental data and the model.

2 $$\begin{aligned} \chi ^2 = \sum _{i} \dfrac{\Big [I^{exp}(\hbar \omega _i) - I(\hbar \omega _i)\Big ]^2}{\sigma _i^2} \end{aligned}$$

Where the summation is carried out over all experimental measurements. $I^{exp}(\hbar \omega _i)$ is the experimental intensity at energy $\hbar \omega _i$ and $I(\hbar \omega _i)$ is the modeled intensity at the same energy. $\sigma _i$ is the intensity uncertainty at each data point. Since the spectra are constructed by the accumulation of single photons, the intensity at each energy naturally follows as Poisson distribution from which the standard deviation on the intensity can be estimated as $\sqrt{n_i+1}$ with $n_i$ the number of photons of energy $\hbar \omega _i$ collected on the detector.

## Supplementary information

Supplementary Information.
