# Supplementary material for: An approach for the measurement of the bulk temperature of single crystal diamond using an X-ray free electron laser
Source: Sci Rep. 2020 Sep 3;10:14564. doi: 10.1038/s41598-020-71350-x (PMC7471281; doi:10.1038/s41598-020-71350-x)
Supplement: Supplementary file 1 — Supplementary Information. [file 41598_2020_71350_MOESM1_ESM.pdf]

Supplemental Material for "An approach for the measurement of the bulk temperature of single crystal diamond using an X-ray Free Electron Laser"

A. Descamps[1,2,*], B. K. Ofori-Okai[1], K. Appel[3], V. Cerantola[3], A. Comley[4], J. H. Eggert[5], L. B. Fletcher[1], D. O. Gericke[6], S. Goede[3], O. Humphries[7], O. Karnbach[7], A. Lazicki[5], R. Loetzsch[8,9], D. McGonegle[7], C. A. J. Palmer[10], C. Plueckthun[3], T. R. Preston[3], R. Redmer[11], D. G. Senesky[2], C. Strohm[3], I. Uschmann[8,9], T. G. White[12], L. Wollenweber[3], G. Monaco[13], J. S. Wark[7], J. B. Hastings[1], U. Zastrau[3], G. Gregori[7], S. H. Glenzer[1], and E. E. McBride[1]

[1]SLAC National Accelerator Laboratory, Menlo Park, 94025, California, USA
[2]Stanford University, Aeronautics and Astronautics department, Stanford, 94305, California, USA
[3]European X-Ray Free-Electron Laser Facility GmbH, Holzkoppel 4, 22869 Schenefeld, Germany
[4]Atomic Weapons Establishment, Aldermaston, Reading, RG7 4PR, UK
[5]Lawrence Livermore National Laboratory, Livermore, California 94550, USA
[6]Centre for Fusion, Space and Astrophysics, Department of Physics, University of Warwick, Coventry CV4 7AL, United Kingdom
[7]Department of Physics, Clarendon Laboratory, Parks Road, University of Oxford, OX1 3PU, UK
[8]Institut für Optik und Quantenelektronik, Friedrich-Schiller-Universität Jena, Max-Wien-Platz 1, D-07743 Jena, Germany
[9]Helmholtz-Institut Jena, Fröbelstieg 3, D-07743 Jena, Germany
[10]School of Mathematics and Physics, Queen's University, University Road BT7 1NN, Belfast, United Kingdom
[11]10 Institut für Physik, Universität Rostock, A.-Einstein-Str. 23-24, D-18059 Rostock, Germany
[12]University of Nevada at Reno, Reno, NV 89506 USA
[13]Dipartimento di Fisica, Universit'a di Trento, via Sommarive 14, 38123 Povo (TN), Italy
[*]adescamp@slac.stanford.edu

ABSTRACT

We present a method to determine the bulk temperature of a single crystal diamond sample at an X-Ray Free Electron Laser using using inelastic X-ray scattering. The experiment was performed at the High Energy Density (HED) instrument at the European XFEL GmbH, Germany. The technique, based on inelastic X-ray scattering and the principle of detailed balance, was demonstrated to give accurate temperature measurements, within 8% for both room temperature diamond and heated diamond to 500 K. Here, the temperature was increased in a controlled way using a resistive heater to test theoretical predictions of the scaling of the signal with temperature. The method was tested by validating the energy of the phonon modes with previous measurements made at room temperature using inelastic X-ray scattering and neutron scattering techniques. This technique could be used to determine the bulk temperature in transient systems with a temporal resolution of 50 fs and for which accurate measurements of thermodynamic properties are vital to build accurate equation of state and transport models.

1 Derivation of the fitting function

The detected scattered intensity, following the data processing described above, is expressed as the sum of the elastic coherent double differential cross section $\left(\dfrac{d^2\sigma}{d\Omega dE'}\right)_{el}$ and the inelastic coherent one-phonon double differential cross section $\left(\dfrac{d^2\sigma}{d\Omega dE'}\right)_{inel}$ [1]. These cross sections correspond to the probability of a photon being scattered elastically and inelastically by the sample in the solid angle Ω with energy between $E^{'}$ and $E^{'}+dE^{'}$.

One can write the instantaneous position, $\vec{R}_s(t)$, of atom s in terms of the normal modes, or phonons,

$$\vec{R}_s(t) = \vec{R}_s^{eq} + \vec{u}_s(t). \tag{1}$$

$\vec{R}_s^{eq}$ represents the equilibrium position of atom s, and $\vec{u}_s(t)$ is a harmonic displacement vector. Using the second quantisation of the vibrational modes in a solid in terms of the phonon creation and annihilation operators[1,2] and assuming thermal equilibrium, $\vec{u}_s(t)$ may be written as,

$$\vec{u}_s(t) = \left(\frac{\hbar}{2M_sN}\right)^{\frac{1}{2}} \sum_{\lambda,\vec{q}} \frac{\vec{\varepsilon}_{\lambda,s}(\vec{q})}{\sqrt{\omega_\lambda(\vec{q})}} \left[\hat{a}_{\lambda,\vec{q}}(t)\exp^{i\vec{q}\cdot\vec{R}_s^{eq}} + \hat{a}^\dagger_{\lambda,\vec{q}}(t)\exp^{-i\vec{q}\cdot\vec{R}_s^{eq}}\right]. \tag{2}$$

In equation (2), M_s is the mass of atom s, and N is the number of nuclei in the crystal. The summation over λ runs over all the phonon branches defined for the crystal (3 acoustic modes and $3(N_b-1)$ optical modes, with N_b the number of atoms in the basis defining the crystal) and all phonon wavevectors, $\vec{q}$, defined within the first Brillouin zone. $\omega_\lambda(\vec{q})$ is the frequency of a phonon characterised by branch λ with a wavevector $\vec{q}$ and a polarisation vector $\vec{\varepsilon}_{\lambda,s}(\vec{q})$ in the first Brillouin zone. In the Heisenberg picture, $\hat{a}_{\lambda,\vec{q}}(t) = \hat{a}_{\lambda,\vec{q}}\exp\{-i\omega_\lambda(\vec{q})t\}$.

1.1 Elastic coherent double differential cross section

The coherent cross section under a perturbation of the atomic position $\vec{R}_s$ can be written

$$\left(\frac{d^2\sigma}{d\Omega dE'}\right)_{coh} = \frac{\sigma_T}{4\pi}\frac{k^{'}}{k}\frac{1}{2\pi\hbar}\sum_{s,s^{'}}\int_{-\infty}^{+\infty}\langle\exp\left\{-i\vec{Q}\cdot\vec{R}_s(0)\right\}\exp\left\{i\vec{Q}\cdot\vec{R}_{s^{'}}(t)\right\}\rangle\exp\{-i\omega t\}dt, \tag{3}$$

Using equations (1), (2), and (3), the elastic coherent cross sections can be expressed as[1]

$$\left(\frac{d^2\sigma}{d\Omega dE'}\right)_{el} = \frac{\sigma_T}{4\pi}N^2\sum_s f_s(Q)^2 e^{-2W_s(Q)}\frac{(2\pi)^3}{V_0}\sum_{\vec{H}}\delta(\vec{Q}-\vec{H})\delta(\hbar\omega), \tag{4}$$

where σ_T is the Thomson coherent cross section for a single electron and $\vec{Q}$ the scattering vector defined as $\vec{k}_{out}-\vec{k}_{in}$ with magnitude Q. V_0 the volume of the unit cell, $\vec{H}$ any reciprocal lattice vector and $\hbar\omega$ the energy exchanged during the scattering event. $f_s(Q)$ is the X-ray atomic form factor for atom s at Q, and $2W_s(Q)$ is the Debye Waller factor associated with the element s. For a cubic crystal, $2W_s(Q)$ is given by

$$\begin{aligned} 2W_s(Q) &= \langle(\vec{Q}\cdot\vec{u}_s)^2\rangle \\ &= \frac{\hbar Q^2}{2M_s}\int_0^{\omega_{s,m}}\frac{Z_s(\omega)}{\omega}\coth\left(\frac{\hbar\omega}{k_BT}\right)d\omega. \end{aligned} \tag{5}$$

$\omega_{m,s}$ is the maximum phonon frequency for element type s, Z_s the normalised phonon density of states for element s. T the temperature of the lattice and k_B the Boltzmann constant.

Given the experimental configuration, equation (4) can be further simplified. First, as the measurement was made on diamond, there is only one element and so s takes only one value. Second, as the measurement was performed on a single crystal and Q was within the first Brillouin zone, $\vec{H}=\vec{0}$. With these considerations, equation (4) becomes

$$\left(\frac{d^2\sigma}{d\Omega dE'}\right)_{el} = I_0\delta(\vec{Q}-\vec{0})\delta(\hbar\omega), \tag{6}$$

and the prefactor I_0 is given by

$$I_0 = \frac{\sigma_T}{4\pi} N^2 f(Q)^2 e^{-2W(Q)} \frac{(2\pi)^3}{V_0}. \tag{7}$$

1.2 Inelastic coherent double differential cross section

Using equations (1), (2), and (3) the inelastic coherent cross sections can be expressed as.

$$\begin{aligned}\left(\frac{d^2\sigma}{d\Omega dE'}\right)_{inel} = &\frac{\sigma_T}{8\pi} N \frac{k_{out}}{k_{in}} \frac{(2\pi)^3}{V_0} \\ &\times \sum_{\lambda} \sum_{\vec{H}} \frac{1}{\omega_\lambda(\vec{q})} \left| \sum_s \frac{f_s(Q)}{\sqrt{M_s}} e^{-W_s(Q)} \left(\vec{Q} \cdot \varepsilon_{\lambda,s}(\vec{q})\right) \right|^2 \\ &\times \left[\langle n(\omega_\lambda(\vec{q})) + 1 \rangle \delta\left(\hbar\omega - \hbar\omega_\lambda(\vec{q})\right) \delta\left(\vec{Q} - \vec{q} - \vec{H}\right) \right. \\ &\quad \left. + \langle n(\omega_\lambda(\vec{q})) \rangle \delta(\hbar\omega + \hbar\omega_\lambda(\vec{q})) \delta\left(\vec{Q} + \vec{q} - \vec{H}\right) \right]\end{aligned} \tag{8}$$

Again, the summation over s runs through all the elements in the unit cell. $\langle n(\omega_\lambda(\vec{q})) \rangle$ is the occupation number of phonons with frequency $\omega_\lambda(\vec{q})$. It is defined as $\langle n(\omega) \rangle = \left[e^{\hbar\omega\beta} - 1\right]^{-1}$ with ω being a real positive number and $\beta = 1/k_B T$.

Given the experimental configuration, equation (8) can be simplified to

$$\begin{aligned}\left(\frac{d^2\sigma}{d\Omega dE'}\right)_{inel} = &\frac{\sigma_T}{8\pi} N \frac{k_{out}}{k_{in}} \frac{(2\pi)^3}{V_0} \\ &\times \sum_{\lambda} \frac{1}{\omega_\lambda(\vec{q})} \left| \frac{f(Q)}{\sqrt{M}} e^{-W(Q)} \left(\vec{Q} \cdot \varepsilon_{\lambda}(\vec{q})\right) \right|^2 \\ &\times \left[\langle n(\omega_\lambda(\vec{q})) + 1 \rangle \delta\left(\hbar\omega - \hbar\omega_\lambda(\vec{q})\right) \delta\left(\vec{Q} - \vec{q}\right) \right. \\ &\quad \left. + \langle n(\omega_\lambda(\vec{q})) \rangle \delta(\hbar\omega + \hbar\omega_\lambda(\vec{q})) \delta\left(\vec{Q} + \vec{q}\right) \right].\end{aligned} \tag{9}$$

Note that in equations (8) and (9), the energy difference $\hbar\omega$ is positive (negative) when the photon loses (gains) energy. However, for the rest of the discussion, we consider $\hbar\omega$ to be positive when the photon gains energy. As the probability of annihilating a phonon of frequency ω_λ and hence transferring energy and momentum to the photon is given by $\langle n(\omega_\lambda) \rangle$, in our convention we should rewrite equation (9) as

$$\left(\frac{d^2\sigma}{d\Omega dE'}\right)_{inel} = \sum_{\lambda} I_\lambda \left[\langle n(\omega_\lambda(\vec{q})) + 1 \rangle \delta\left(\hbar\omega + \hbar\omega_\lambda(\vec{q})\right) \delta\left(\vec{Q} + \vec{q}\right) + \langle n(\omega_\lambda(\vec{q})) \rangle \delta(\hbar\omega - \hbar\omega_\lambda(\vec{q})) \delta\left(\vec{Q} - \vec{q}\right) \right], \tag{10}$$

where the prefactors have been collected into a single term I_λ given by

$$I_\lambda = \frac{\sigma_T}{8\pi} N \frac{k_{out}}{k_{in}} \frac{(2\pi)^3}{V_0} \frac{1}{\omega_\lambda(\vec{q})} \left| \frac{f(Q)}{\sqrt{M}} e^{-W(Q)} \left(\vec{Q} \cdot \varepsilon_\lambda(\vec{q})\right) \right|^2. \tag{11}$$

In equation (10), the factors $\langle n(\omega_\lambda(\vec{q})) + 1 \rangle$ and $\langle n(\omega_\lambda(\vec{q})) \rangle$ are defined for positive frequency $\omega_\lambda(\vec{q})$. The equation can be re-written with the function $\langle n(\omega) \rangle$ defined for positive and negative frequencies. To do this, we first utilise the definition of $\langle n(\omega) \rangle$

$$
\begin{aligned}
\langle n(\omega_\lambda(\vec{q}))+1\rangle &= \left[e^{\hbar\omega_\lambda(\vec{q})\beta}-1\right]^{-1}+1 \\
&= \frac{e^{\hbar\omega_\lambda(\vec{q})\beta}}{e^{\hbar\omega_\lambda(\vec{q})\beta}-1} \\
&= \frac{1}{e^{-\hbar\omega_\lambda(\vec{q})\beta}}\frac{1}{e^{\hbar\omega_\lambda(\vec{q})\beta}-1} \\
&= \frac{1}{1-e^{-\hbar\omega_\lambda(\vec{q})\beta}} \\
&= -\frac{1}{e^{-\hbar\omega_\lambda(\vec{q})\beta}-1} \\
&= -\langle n(-\omega_\lambda(\vec{q}))\rangle
\end{aligned} \tag{12}
$$

Using this we can express equation (10) as

$$
\left(\frac{d^2\sigma}{d\Omega dE'}\right)_{inel} = \sum_\lambda I_\lambda \Big[-\langle n(-\omega_\lambda(\vec{q}))\rangle \delta(\hbar\omega+\hbar\omega_\lambda(\vec{q}))\delta(\vec{Q}+\vec{q}) + \langle n(\omega_\lambda(\vec{q}))\rangle \delta(\hbar\omega-\hbar\omega_\lambda(\vec{q}))\delta(\vec{Q}-\vec{q})\Big] \tag{13}
$$

Next, we use the sampling property of the Dirac delta function to write

$$
\langle n(\omega_\lambda(\vec{q}))\rangle \delta(\hbar\omega-\hbar\omega_\lambda(\vec{q})) = \langle n(\omega)\rangle \delta(\hbar\omega-\hbar\omega_\lambda(\vec{q})), \tag{14a}
$$
$$
\langle n(-\omega_\lambda(\vec{q}))\rangle \delta(\hbar\omega+\hbar\omega_\lambda(\vec{q})) = \langle n(\omega)\rangle \delta(\hbar\omega+\hbar\omega_\lambda(\vec{q})). \tag{14b}
$$

These substitutions replace the values $\langle n(\omega_\lambda(\vec{q}))\rangle$ with the function $\langle n(\omega)\rangle$ which is independent of λ and hence can be taken out of the sum.

$$
\left(\frac{d^2\sigma}{d\Omega dE'}\right)_{inel} = \langle n(\omega)\rangle \sum_\lambda I_\lambda \Big[-\delta(\hbar\omega+\hbar\omega_\lambda(\vec{q}))\delta(\vec{Q}+\vec{q}) + \delta(\hbar\omega-\hbar\omega_\lambda(\vec{q}))\delta(\vec{Q}-\vec{q})\Big]. \tag{15}
$$

The previous derivation assumed that the displacement vector $\vec{u}_s(t)$ is caused by harmonic forces. In reality, non-harmonic effects such as phonon-phonon interactions that may arise from terms beyond the second order in the Taylor expansion of the interatomic potential could play a role in the measured intensity. To account for such effects, the δ-Dirac function are replaced by a Lorentzian lineshape describing the damping of the phonon mode through non harmonic effects[2], as

$$
\left(\frac{d^2\sigma}{d\Omega dE'}\right)_{inel} = \langle n(\omega)\rangle \sum_\lambda \frac{I_\lambda}{\pi}\left[-\frac{\Gamma_\lambda(\vec{q})}{\left(\hbar\omega+\hbar\omega_\lambda(\vec{q})\right)^2+\Gamma_\lambda(\vec{q})^2}\delta(\vec{Q}+\vec{q}) + \frac{\Gamma_\lambda(\vec{q})}{\left(\hbar\omega-\hbar\omega_\lambda(\vec{q})\right)^2+\Gamma_\lambda(\vec{q})^2}\delta(\vec{Q}-\vec{q})\right] \tag{16}
$$

Finally equations (6) and (16) are used to define the fitting function used to analyse the experimental data

$$
\begin{aligned}
I(\hbar\omega) = R(\hbar\omega,\vec{q}) * \Bigg(&\frac{I_0}{\pi}\frac{\Gamma_0(\vec{q})}{(\hbar\omega)^2+\Gamma_0(\vec{q})^2} \\
&+ \langle n(\omega)\rangle \sum_\lambda \frac{I_\lambda}{\pi}\left[-\frac{\Gamma_\lambda(\vec{q})}{\left(\hbar\omega+\hbar\omega_\lambda(\vec{q})\right)^2+\Gamma_\lambda(\vec{q})^2} + \frac{\Gamma_\lambda(\vec{q})}{\left(\hbar\omega-\hbar\omega_\lambda(\vec{q})\right)^2+\Gamma_\lambda(\vec{q})^2}\right]\Bigg).
\end{aligned} \tag{17}
$$

$R(\hbar\omega,\vec{q})$ is the instrument function as determined from the spectra obtained from 500 μm of PMMA for room temperature, and 250 μm SiO_2 for the heated case. In equation 17, T, I_0, I_λ, $\Gamma_\lambda(\vec{q})$, Γ_0, $\omega_\lambda(\vec{q})$ are kept as free parameters.

2 Determination of the energy resolution of the instrument

The inelastic scattering spectra used to determine the instrument function for the room temperature and heated (500 K) diamond measurements are shown in figure 1. The top row corresponds to the energy resolution as measured from the quasi-elastic scattering of 500 μm-thick PMMA sample for the left (a), centre (b) and right analyser (c). The bottom row shows the measurement for the heated case using 250 μm-thick SiO_2 sample. The energy resolution is defined as the Full Width at Half Maximum of the fitted Voigt profile (solid black line) to the recorded intensity. The vertical dashed lines correspond to the edges of the analyser and are described in the main manuscript.

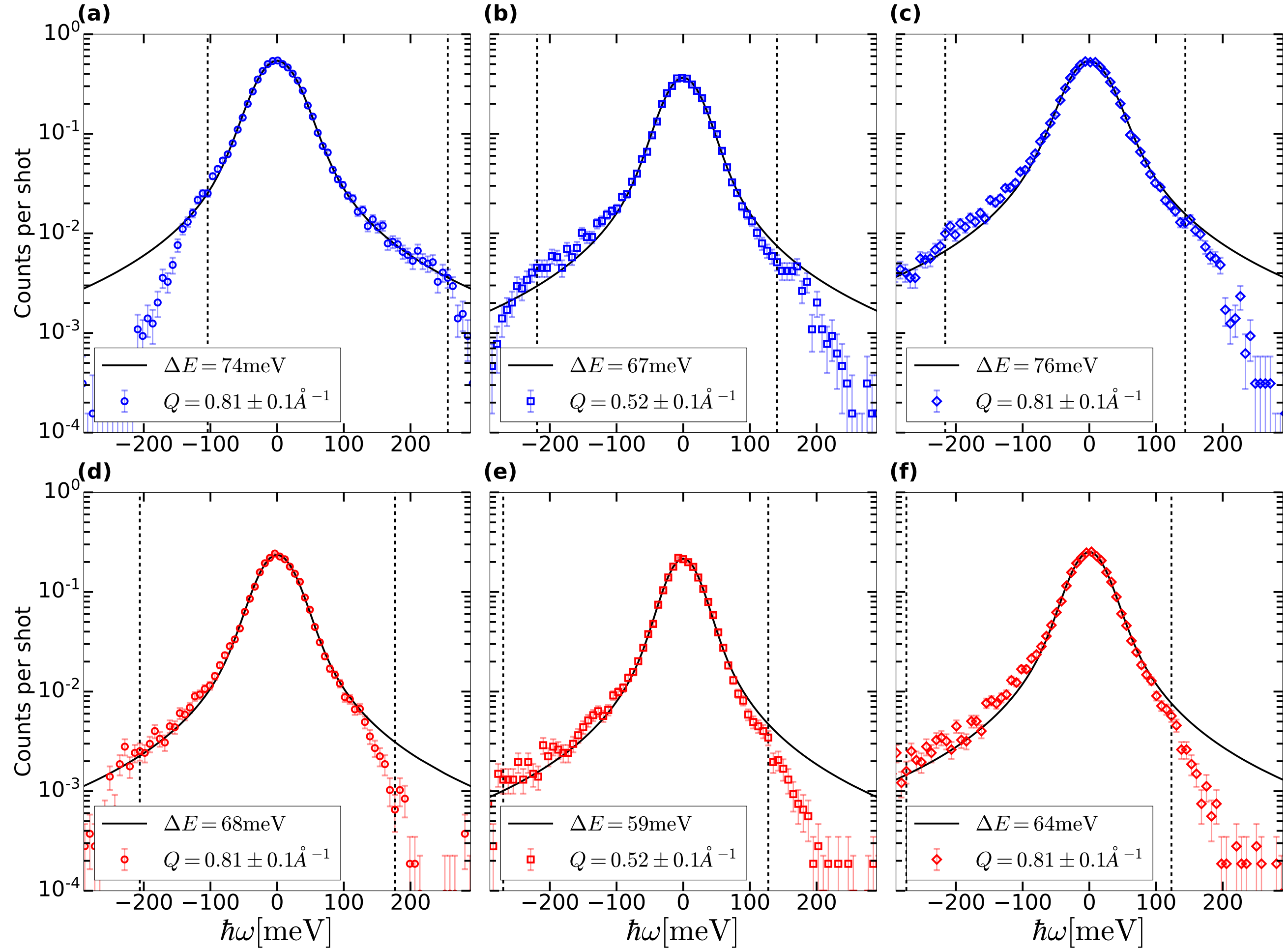

Figure 1. Inelastic spectra for each analyser used to determine the instrument function $R(\hbar\omega, \vec{q})$ for the cold measurement on 500 μm-thick PMMA (a-c) and the heated (500 K) measurement on 250 μm-thick SiO_2 (d-f). The solid black line represents the best Voigt profile fit to the experimental data from which the energy resolution ΔE is defined as the Full Width at Half Maximum.

3 Experimental results

Inelastic X-ray scattering spectra recorded on the centre analyser for room temperature diamond. The experimental data and the best fit (solid black line) are identical to the ones presented in figure 3 (b) in the main manuscript. As discussed in the main manuscript, the quasi-elastic component is not visible since the measurement was performed on high purity, single crystal diamond, see $I_0 \times \Gamma_0$ values reported in table 2. The dot-dashed lines correspond to the Stokes and anti-Stokes components extracted for the best fit parameters reported in table 2. The asymmetry of these features is due to the asymmetry of the Bose-Einstein distribution.

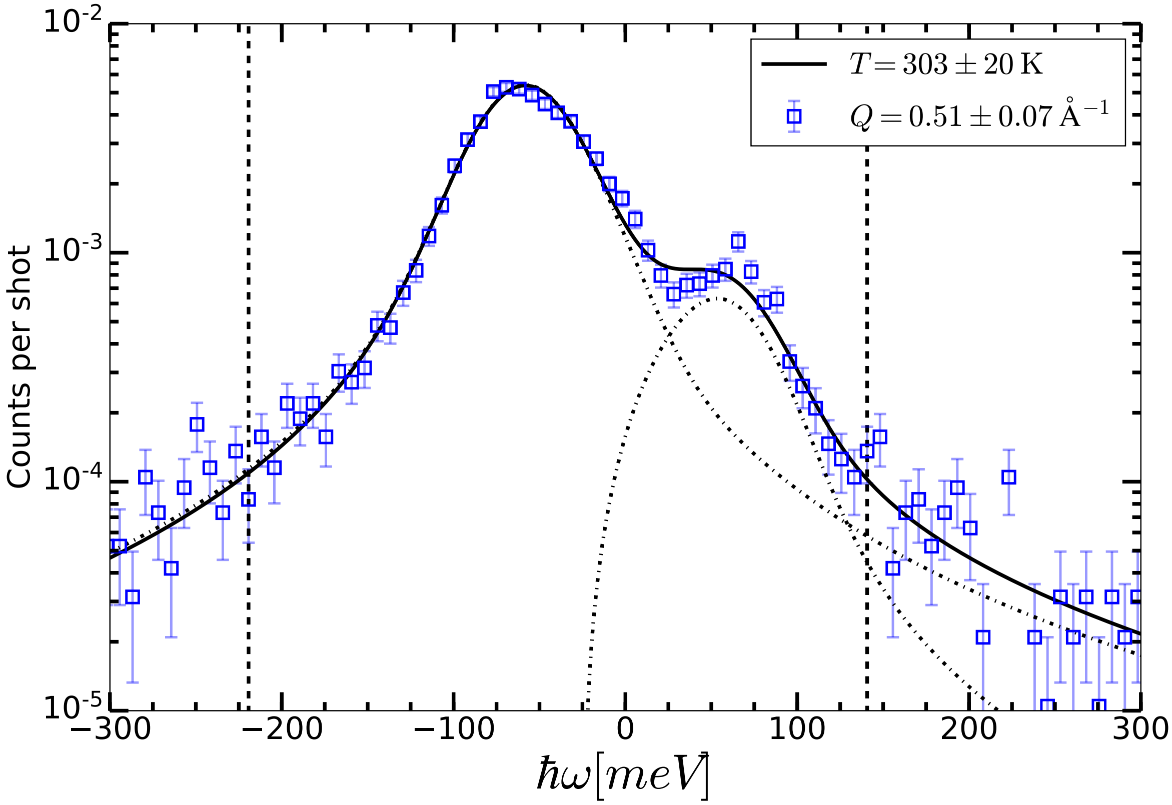

Figure 2. Inelastic X-ray scattering data collected at room temperature on the centre analyser. The experimental data (blue squares) are shown along with the best fit using equation 1 see main manuscript (solid black line). The dot-dashed lines correspond to the inelastic components of the spectrum. The quasi-elastic component is not visible since its contribution is negligible due the single crystal nature of the sample. The asymmetry of the inelastic peaks is caused by the Bose-Einstein distribution. It should be noted that the experimental data are fitted directly using equation 1 (main manuscript), solid black curve, and then the Stokes and anti-Stokes features are then individually extracted after the fitting procedure.

References

1. Squires, G. L. *Correlation functions in nuclear scattering*, 61–85 (Cambridge University Press, 2012), 3 edn.

2. Marshall, W. & Lovesey, S. W. *Scattering by phonons*, 64–93 (Oxford University Press, 1971).
